# Local Ablative Strategies for Ductal Pancreatic Cancer (Radiofrequency Ablation, Irreversible Electroporation): A Review

**DOI:** 10.1155/2016/4508376

**Published:** 2016-02-15

**Authors:** Salvatore Paiella, Roberto Salvia, Marco Ramera, Roberto Girelli, Isabella Frigerio, Alessandro Giardino, Valentina Allegrini, Claudio Bassi

**Affiliations:** ^1^Unit of General Surgery B, The Pancreas Institute, G.B. Rossi Hospital, University of Verona Hospital Trust, Verona, Italy; ^2^Pancreatic Surgical Unit, Casa di Cura Pederzoli, Peschiera del Garda, Verona, Italy

## Abstract

Pancreatic ductal adenocarcinoma (PDAC) has still a dismal prognosis. Locally advanced pancreatic cancer (LAPC) accounts for the 40% of the new diagnoses. Current treatment options are based on chemo- and radiotherapy regimens. Local ablative techniques seem to be the future therapeutic option for stage-III patients with PDAC. Radiofrequency Ablation (RFA) and Irreversible Electroporation (IRE) are actually the most emerging local ablative techniques used on LAPC. Initial clinical studies on the use of these techniques have already demonstrated encouraging results in terms of safety and feasibility. Unfortunately, few studies on their efficacy are currently available. Even though some reports on the overall survival are encouraging, randomized studies are still required to corroborate these findings. This study provides an up-to-date overview and a thematic summary of the current available evidence on the application of RFA and IRE on PDAC, together with a comparison of the two procedures.

## 1. Introduction

PDAC is one of the deadliest cancer types. It accounts for about 7% of all cancer deaths and is actually the fourth cause of cancer death in the United States [1]. Only 20% of PDAC are resectable at time of diagnosis (with a 5-year survival of less than 20%), while the majority of patients are candidates only for chemotherapy or chemoradiotherapy according to various protocols [2–4]. 40% of patients are diagnosed with a locally advanced disease, with few chances to undergo surgery even after neoadjuvant treatments. Median overall survival (OS) reported for patients treated with upfront surgery and adjuvant therapy is about 20–22 months [5–7], while it is about 9.2–11.7 months for stage-III locally advanced pancreatic cancer (LAPC) treated with Gemcitabine alone [8–10] and 9–13 months for patients treated with chemo(radio)therapy [11]. Given that LAPC is nearly the most frequent diagnosis to face and that downstaging occurs only in 10–20% of patients [12], the novel local therapies, such as Radiofrequency Ablation (RFA) and Irreversible Electroporation (IRE), have been proposed as new treatment options in the multimodal treatment of the disease [13]. The aim of this paper is to evaluate and compare technical aspects, indications, and results of the application of both RFA and IRE on LAPC.

## 2. Physical Bases and Principles of Techniques

Local thermal or nonthermal techniques are applied to ultimately induce irreversible cellular damage leading to cell death* via* either apoptosis or coagulative necrosis. Physical bases and principles of technique of both RFA and IRE are briefly shown below.

### 2.1. RFA

**Figure 1:**
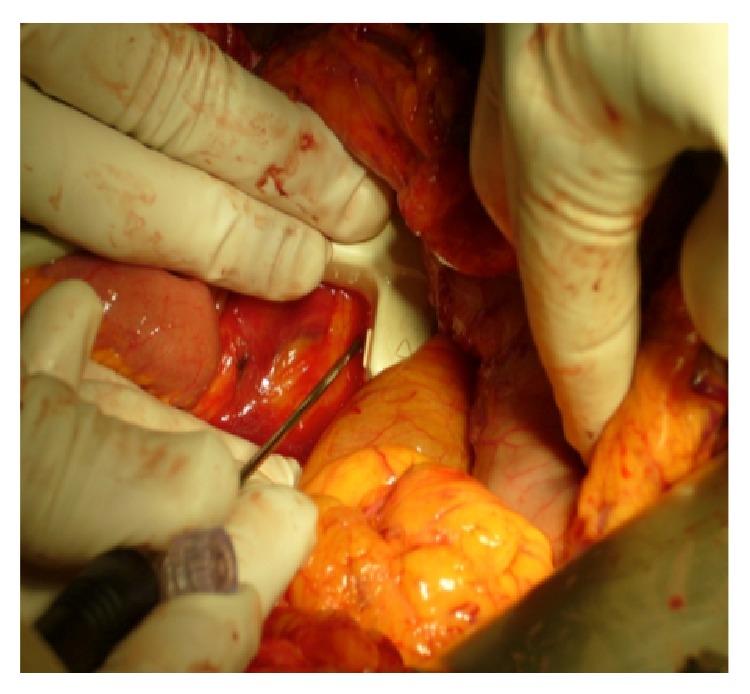
US-guided intraoperative application of RFA tip.

RFA is an ablative therapy that, through the application of a high-frequency alternating current, conveyed by one or more needle electrodes, generates local high temperatures, leading to coagulative necrosis and protein denaturation inside neoplastic tissue. While at temperatures between 60 and 100°C immediate coagulation of tissue is induced, with irreversible damage to the inner structure of the cells, using 100–110°C, the tissue vaporizes and carbonizes [14]. At the beginning of the application of RFA on PDAC, high morbidity (0–40%) and mortality (0–25%) rates have been reported [15]. Later,* ex vivo* studies demonstrated that an adjustment of both temperature and length of the dispensed energy would conduce to better outcomes with fewer complications [16, 17]. Although several temperatures have been used, ranging from less than 30°C to 90°C according to the equipment used to perform RFA [18, 19], it seems that the ideal parameters to consider are actually represented by 90°C for 5 minutes, with a distance of 10 and 15 mm between probe and duodenum and portomesenteric axis, respectively [20]. The electrode must be introduced inside the tumor under ultrasound or CT-guidance and the procedure can be monitored in real time by ultrasound with a safe distance of the RFA probe from duodenum or portomesenteric vessels of 5–10 mm (Figure 1). The procedure can either be performed through laparotomy, percutaneously, or through an endoscopic approach [21, 22]. These mini-invasive techniques could be useful to avoid laparotomy, in patients unsuitable for surgery or in case of LAPC of the body-tail of the pancreas without symptoms.

### 2.2. IRE

IRE is a nonthermal technique that induces cell death. The ablative effect is based on the delivery of short high-voltage electric current fields that induce cell death. The application of short high-voltage electric pulses, conveyed by one or more monopolar electrodes, causes the irreversible permeabilization of the lipid bilayer, the disruption of intracellular homeostasis, and the activation of apoptotic pathways, ultimately resulting in cell death of neoplastic cells [30–36]. Interestingly, and differently from RFA, IRE is able to preserve surrounding structures, such as the underlying matrix that can work again as a scaffold for the healing tissue, or the vital structures like nerves or vessels [37–39]. Narayanan et al. in a retrospective review of 101 IRE procedures performed on different organs for tumors abutting or encasing major vessels reported a rate of vascular changes of only 4.4% (thrombosis or mild vascular narrowing phenomena) demonstrating a very high rate of patency of the major vessels in humans, after the application of IRE [40]. The proper ability of IRE to preserve the vessels could be a fundamental aspect when the tumor encases the major peripancreatic vessels, when the application of RFA could result as difficult, dangerous, and inefficacious (because of the heat-sink effect). However, it has been advocated that the cellular damage induced by IRE could be partially thermal. In fact, in some conditions of high intensity, current applied IRE can produce a coagulative necrosis similar to the one produced by thermal techniques [41]. Dunki-Jacobs et al. further investigated this aspect, concluding that IRE does not produce significant thermal energy, at least using the settings most commonly applied in clinical treatment. On the other side, they demonstrated that the presence of metallic stent could increase the risk of producing thermal injuries, because of the conductivity of the metal [42]. This aspect might be important in those patients carrying a biliary metallic stent for jaundice palliation. Hence, it should be kept in mind that IRE is not a “pure” nonthermal technique and that it remains connected in some way with thermal effects. Treatment planning of IRE is of utmost importance and several tools are available to properly manage the application of the technique [43–45]. Martin accurately described the procedure with the ideal settings on pancreas [46, 47].

## 3. Indications and Contraindications

Preoperative work-up should always include routine laboratory tests (including CA 19-9 levels) and a 3-phase CT-scan of the abdomen in order to assess exactly the location and the dimension of the tumor, the type of vascular infiltration, and the possible presence of abdominal metastases. Local ablative therapies, such as RFA and IRE, should be allotted to those tumors that show a local growth pattern without systemic involvement and should be considered as consolidative therapies in the multimodal therapeutic approach to LAPC. The decision to perform one or the other should be taken by a multidisciplinary group, considering the patients' comorbidities and quality of life, the natural history of the tumor, and, mostly, the response to medical oncological treatments. The assessment of resectability of LAPC after neoadjuvant therapy is still difficult [48]. In the FOLFIRINOX era, imaging seems to have no longer ability in determining the real response rate after neoadjuvant therapy [49]. In the future, RFA and IRE will be applied more often as “salvage” cytoreductive therapies or in the context of properly designed clinical trials, at least until randomized controlled trials will not demonstrate their oncological efficacy. Furthermore, it is of paramount importance that RFA and IRE should be performed selectively in high-volume HPB centers, and, for percutaneous-only approaches, by experienced interventional radiologists.

### 3.1. RFA

Indications are as follows. The most common worldwide application of RFA on PDAC is represented by the treatment of stage-III patients, either in case of no further response to standard systemic treatments or as an upfront option at the time of diagnosis [15, 16, 18, 28, 50–58]. However, some studies included also stage-IV metastatic patients [18, 19, 59, 60], probably to induce a positive modulation of the immune system [61]. Recently, the application of RFA upfront has been justified on the basis of a presumed immunological antitumoral stimulation aroused by RFA [50]; a randomized controlled study to prove or to disown it is currently running. However, RFA should be considered as a new tool in the surgeon's toolbox, in the context of a multidisciplinary approach to PDAC.

Tumor diameter is not a crucial parameter in the evaluation of the application of RFA as the technique itself allows ablating up to 5 cm or more [62]. Unfortunately, because of the proximity of vital structures surrounding PDAC (infiltrated by definition in LAPC), the whole ablation of the tumor would result in being too risky. Then, it is preferred to treat the biggest possible area, performing also pull-backs of the tip, leaving a “security ring” at the periphery of the tumor in order to avoid thermal injuries of the nearby structures [63]. This viable tissue at the periphery of the tumor will later be the target of the radiotherapy, to complete the ablation of the tumor [17].

Contraindications are as follows. RFA can interfere with implanted pacemakers and cardioverter defibrillators due to electromagnetic energy [64]. Hence, a cardiac evaluation is recommended in this special subset of patients, for a possible resynchronization of these devices.

### 3.2. IRE

**Figure 2:**
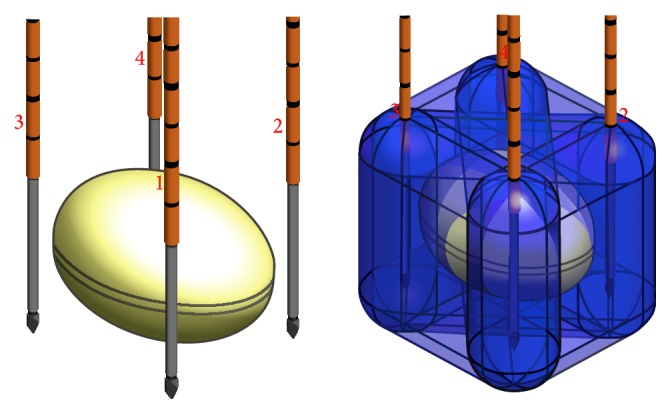
Example of a computerized model of the application of a 4-needle IRE technique. The yellow oval represents the tumor. Crossing blue beams represent the energy developed between each couple of probes.

Indications are as follows. Almost all the applications of IRE on PDAC are on stage-III LAPC [23, 24, 27, 47, 65–71]. Narayanan et al. reported three cases of application of IRE on stage-IV patients with centimetric liver metastases from PDAC and two cases of application of IRE as a “bridge” therapy in LAPC before submitting the patients successfully to a radical surgical resection [72]. Simultaneously, some papers report promising results on the use of IRE for margin accentuation, as a technique to reduce the rate of R1 resections in case of locally advanced/borderline resectable PDAC [24, 65, 68, 73]. In general, IRE works better on tumor sizes that are 3 to 3.5 cm and it is important to plan the ablation technique properly (Figure 2) in order to treat the whole tumor [74]. In addition, the application of IRE seems to be more appropriate than RFA when the tumor encapsulates the superior mesenteric artery. In fact, the application of multiple needles allows bracketing the artery and treating. Furthermore, the negligible amount of heat associated with IRE allows safe and efficacious ablations.

Contraindications are as follows. In general, electric fields applied to human body can cause arrhythmias; hence, it is of utmost importance to reduce this risk synchronizing pulsing with the heart rhythm, using a dedicated device [75]. For these reasons, IRE is contraindicated in patients with pacemakers or with cardiac arrhythmias. Moreover, a metallic biliary stent should be removed intraoperatively before IRE, because the presence of the metal could increase the risk of thermal injury [70].

## 4. Oncological Outcomes

All the results regarding the oncological outcomes of the application of RFA and IRE on PDAC are biased by the nature itself of the studies. The reports include very heterogeneous populations of patients, with either stage-III or stage-IV disease. There are no randomized controlled studies available. Most of them were created as phase-I studies in order to demonstrate the safety of the techniques; then, oncological outcomes were only secondary goals. Despite these intrinsic problems, some encouraging results can be extracted.

### 4.1. RFA

Given that all patients treated with RFA will relentlessly progress [16, 53, 57, 60], some papers report good oncological results obtained with the use of RFA on PDAC. Spiliotis et al. reported a reassuring mean survival of 30 months for patients suffering from PDAC treated with RFA, compared to the 13 months' survival for patients receiving a standard systemic treatment (*p* = 0.0048) [18]. Giardino et al. cited a median overall survival (OS) for their whole series (*n* = 107) of 25.6 months, 14.7 months in the group of patients receiving RFA plus several possible systemic treatments, and 25.6 months in the group treated with primary treatments plus RFA plus further systemic treatments (*p* = 0.004). Interestingly, those patients who received this latter therapy, the so-called “triple approach strategy,” with RFA plus radiochemotherapy plus intra-arterial chemotherapy with further systemic treatments, had an OS of 34.0 months [17].

### 4.2. IRE

Despite the increasing number of papers reporting the application of IRE on PDAC, none of these studies is designed to demonstrate the oncological efficacy of the procedure. In fact, they mostly deal with safety and feasibility issues and for this reason the populations considered are not ideal models for the analysis of oncological outcomes.  Table 1 shows the studies reporting data on the efficacy of IRE; however, all these results must be considered cautiously. Interestingly, two papers described five cases of downstaging with R0-resections of LAPC treated with percutaneous IRE [26, 27].

A recent paper from Martin et al. reports an outstanding median OS of 24.9 months (range 12.4–85 months; *n* = 200), for patients treated with IRE in situ or pancreatic resections with major vascular resections and IRE for the margin accentuation, after 6 months (median) of induction chemotherapy or chemo(radio)therapy [24]. As the authors state in the paper, the population considered is made of highly selected patients and this represents an important selection bias. However, these results are very surprising and encouraging, especially if compared with the historical populations of patients reported in literature suffering from LAPC.

Recently, Philips et al. reported an increased risk of accelerating the tumor growth after the application of incomplete sessions of IRE in a murine model. This worrisome finding should be further clarified and possibly verified in clinical scenarios [76].

## 5. Complications

The majority of the complications caused by local ablative techniques are consequence of an uncontrolled heating of the structures surrounding the tumor, rather than a direct lesion caused by the tip of the probe used. Therefore, obviously, it is of paramount importance to plan properly the procedure, setting the parameters according to location, dimensions, and morphology of the tumor.

### 5.1. RFA

The first clinical applications of RFA were afflicted by a high rate of morbidity and mortality, ranging from 0 to 40% and from 0 to 25%, respectively [15]. Once the temperature was lowered from 105 to 90°C for 5 minutes' length, the reported number of complications reduced in parallel [16, 17]. The deaths related to RFA were most commonly caused by gastrointestinal hemorrhages. The most recent cohort of patients treated with RFA comes again from Girelli et al. They reported a reduction of the morbidity rate to 8%, with a mortality rate of 0% [50]. The overall reported rates of RFA-related complications and RFA-related mortality are 13.6 and 1.5%, respectively [13]. The most common complications reported in literature are gastrointestinal hemorrhages and minor local bleedings, acute pancreatitis (mild or severe), pancreatic and biliary fistulas, duodenal injury (thermal or direct), and portal vein thrombosis. It is suggested to cool the duodenum during the procedure with a cold saline solution administered using the nasogastric tube, to preserve it from the possible thermal injury [20].

### 5.2. IRE

A recent systematic review reported an IRE-related complication rate of 13%, with an IRE-related mortality of 2% [13]. The overall reported complications rate of the percutaneous approach is 29% [77]. Martin et al., in a recent study with a population of 200 patients suffering from LAPC treated with IRE, showed an overall rate of adverse events of 37% (74 patients with 149 overall complications) and a mortality rate of 2% [24]. The largest single-center percutaneous series of 50 IRE described an overall number of 27 complications [26]. The most common complications (including both percutaneous and open techniques) described after the use of IRE on pancreas are pancreatitis, pneumothorax, hematoma, abdominal pain, bile leakage, pancreatic leakage, duodenal leakage, duodenal ulcer, and deep vein thrombosis.

## 6. Ablative Techniques and Imaging

One of the most interesting and useful aspects of the application of the ablative techniques on PDAC is the possibility to appraise the amount of tissue ablated and the relationship between the treated area and tumor margins.

### 6.1. RFA

**Figure 3:**
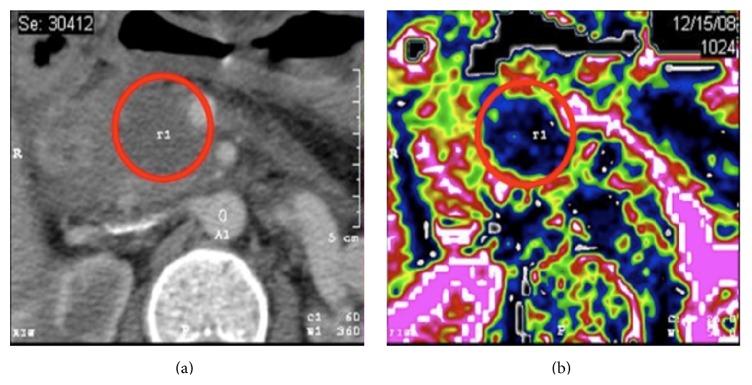
(a) Preoperative CT-scan of a locally advanced pancreatic cancer. (b) Post-RFA perfusion CT-scan, showing a postablative area of decreased perfusion within the head of the pancreas. Copyright Chirurgia del Pancreas Verona.

**Figure 4:**
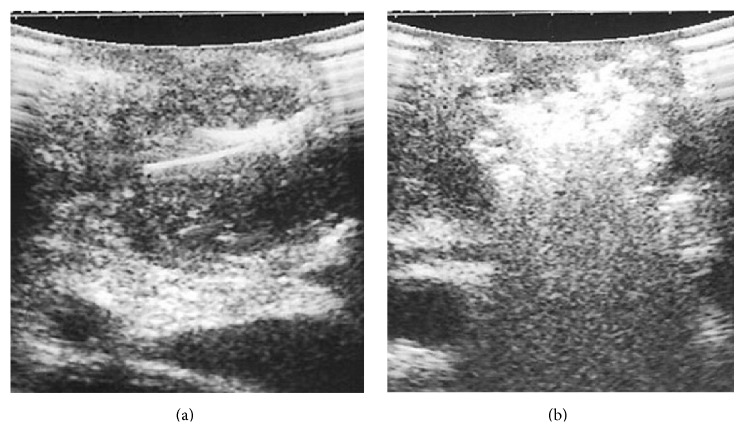
(a) The tip for RFA is placed inside the tumor under US-guidance. (b) During RFA, the lesion becomes immediately hyperechoic.

For RFA, and in general for “thermal techniques,” the gold standard of imaging is represented by cross-sectional imaging* via* helical CT-scan, rather than ultrasonography [78]. A postablative hypointense area can be observed as result of the treatment (Figure 3). At our institution, we perform a three-phase contrast-enhanced CT-scan of the abdomen at postoperative days 7 and 30. During the procedure, ultrasonography can both guide the tip and detect the immediate results of the thermal damage (Figure 4).

### 6.2. IRE

Because of the nonthermal noncoagulative action of IRE and because of the consequent preservation of the vessels, the application of contrast-enhanced CT-scan after IRE would not have the same results as for RFA. Several techniques have been used to evaluate the effect of the application of IRE. Magnetic resonance electrical impedance tomography (MREIT) seems to be able to identify the areas with insufficient electric field, in order to label potential untreated zones after IRE [79]. Either contrast-enhanced or diffusion weighted MRI seems to be able to depict the tissue zones ablated with IRE [80–82]. Even if using a swine model, a recent study stated that the best identification of tissue ablation after IRE is obtained with portal vein phase CT-scan. Anyway, differently from RFA, during CT-scan, a contrast enhancement can be appreciated on the more delayed venous phase, due to the congestion of blood in the tumor vessels [32, 83, 84]. Ultrasound findings during and after IRE could be useful to evaluate the approximate area of ablation. In the acute phase, a hypoechoic area can be registered, with a hyperechoic external rim that forms 90–120 minutes after the treatment [85, 86]. However, Martin et al. state that an early postoperative scanning after IRE should be performed only to rule out possible complications (deep vein thrombosis) and not to evaluate ablation efficacy [24]. It still has to be evaluated if a serum CA 19-9 level decrease could be used as predictor of efficacy.

## 7. Ablative Techniques and Immune System

The strongest factor supporting the clinical application of the ablative techniques, especially of RFA, is represented by their positive antitumoral effect on immune system. Nowadays, thanks to the several studies that have been published, RFA is called prudently “endogenous vaccine” for PDAC. How strong is this power and which are the best timing and proper methods to use it remain to be established.

### 7.1. RFA

All the processes involving the modulation of immune system have been exhaustively described by Chu and Dupuy [87]. While the direct effect of RFA is clearly represented by the necrotic area immediately identifiable after the procedure, on the other hand, the indirect effects are on the viable zone adjacent to this area (transition or peripheral zone). The cells populating the peripheral zone are affected by the RFA in terms of alteration of metabolic endocellular processes that makes them quite sensible to further cytolytic therapies, such as chemo- or chemoradiotherapy. These effects result, ultimately, in the almost total destruction of the tumor. In parallel to this “local” action, RFA can cause a “systemic” immune response involving proinflammatory cytokines [88–90], lymphocytes (T-, B-, and NK-types) [91–94], and antibodies [95] that are responsible for acquired antitumoral antigen-specific immunity [96, 97] that could confer better survival in some patients treated with RFA. It also seems promising to use the synergic use of RFA together with topic specific cytolytic agents or with immunotherapy with monoclonal antibodies or vaccine [98]. However, most of the findings described come from experimental models or from* in vivo* results from organs other than pancreas. Of course, there is need for more preclinical models, investigational studies, and large randomized controlled clinical trials to demonstrate the effects of RFA on PDAC selectively.

### 7.2. IRE

The immune system involvement after IRE has not been thoroughly investigated yet.

Some reports support the evidence that since the proteins are not denatured in IRE (differently from in RFA), in theory, this could result in a weak specific antigenic stimulation against the tumor. In fact, Al-Sakere et al., using murine models of sarcoma treated with IRE, showed that there is no local infiltration of tumor cells among the treated tissue. An early and prolonged decrease in both T lymphocytes (both CD4+ and CD8+) and antigen presenting cells can be detected within a couple of hours after IRE [99]. As they support, this is a demonstration that IRE does not need the involvement of the immune system to kill neoplastic cells and, for this reason, it could be applied on immunosuppressed patients too. On the other hand, other reports reached the evidence that both local and systemic immune antitumoral stimulation are enhanced after IRE [100, 101]. This aspect could be referred to the peculiar type of cellular death caused by IRE: the activation of the apoptotic processes leads to the release of intact and stimulating endogenous tumoral antigens able to induce a strong global antitumoral activity. Ultimately, according to Neal II et al., IRE would be able to generate the “three signals' sequence” that is mandatory for the production of a cytotoxic T-cell response [101].

These conflicting reports demonstrate how far we are from the understanding of the exact involvement of the immune system and how much we need further preclinical and clinical models.

## 8. Conclusions

RFA and IRE represent an innovation on the multimodal treatment of LAPC. The undeniable advantages connected with the use of these techniques are represented by low morbidity, reduced costs, possible percutaneous application, almost selective action with preservation of peritumoral tissues, possible application to patients at a high-risk for surgery, and suspected positive immune stimulation. Moreover, taking into account their positive effect on the immune system, they could be potentially very useful in those patients that, somehow, show an indolent disease, with a prevalent local growth and without a wide systemic involvement.

Nevertheless, as for any other technology introduced in medical practice, RFA and IRE have to be evaluated prospectively and systematically according to the IDEAL framework for evaluation of surgical innovation [102]. In the IDEAL paradigm for the introduction of new technologies in surgery, the application of RFA and IRE on LAPC is still stuck on the 2a phase where few people still adopt the technique, where the patients are selected, where the outcomes are mostly safety and feasibility, and where the clinical outcomes are timidly reported. Hence, the current available evidence is still not sufficient to permit conclusions about long-term benefits.

Nowadays, the patients suffering from LAPC are still waiting for answers that medical oncologists cannot give. Surgery and new ablative technologies can play an important role in giving hope, prolonging survival, and improving quality of life of the patients suffering from LAPC. However, we must move toward a rigorous evaluation of these new procedures through the creation of appropriate randomized controlled studies.

## Figures and Tables

**Table 1:** Efficacy of IRE on PDAC.

Author	Number of patients	Approach	Type of study	Survival (mo.)
Martin et al. [23]	54	Open (52) Percutaneously (2)	Propensity-matched comparison with standard chemo- or chemoradiation	20.2

Martin et al. [24]	200	Open	Data from multicenter registry	24.9

Trueba-Arguiñarena et al. [25]	1	Percutaneously	Case report	f-up 12 mo.

Narayanan et al. [26]	43	Percutaneously	Prospective	16.2

Belfiore et al. [27]	20	Percutaneously	Retrospective	12.9

Pai et al. [28]	5	Percutaneously	Phase-1 safety and feasibility	Range 1–6 mo.

Paiella et al. [29]	10	Open	Phase-1 safety and feasibility	Median 6.4, range 2.9–15.9
